# Protocol for accurately annotating circular RNA and microRNA in giant vertebrate genomes

**DOI:** 10.1016/j.xpro.2025.103912

**Published:** 2025-06-20

**Authors:** Ketan Mishra

**Affiliations:** 1 Department of Cell and Molecular Biology, Karolinska Institute, 171 65 Stockholm, Sweden

**Keywords:** Bioinformatics, Sequence analysis, Genetics, Genomics, Sequencing, RNA-seq, Gene Expression

## Abstract

With the advent of long-read sequencing techniques, there has been an increase in the sequencing and assembly of giant genomes such as salamanders and lungfish. Here, I present a protocol for accurately annotating non-coding RNAs, specifically circular RNAs and microRNAs, using genomic and transcriptomic data. I describe quality checks and *post hoc* steps to minimize false-positive predictions in repeat element-rich genomes. This protocol has potential application for annotating non-coding RNAs in other giant vertebrate genomes.

For complete details on the use and execution of this protocol, please refer to Brown et al.^1^

## Highlights

• Stepwise framework for annotating circular RNA and microRNA
• Procedures for adapting existing tools for giant vertebrate genomes
• Detailed quality checks for limiting false-positive predictions

## Abstract (editor highlights)

Publisher’s note: Undertaking any experimental protocol requires adherence to local institutional guidelines for laboratory safety and ethics.

## Graphical abstract

**Figure:**
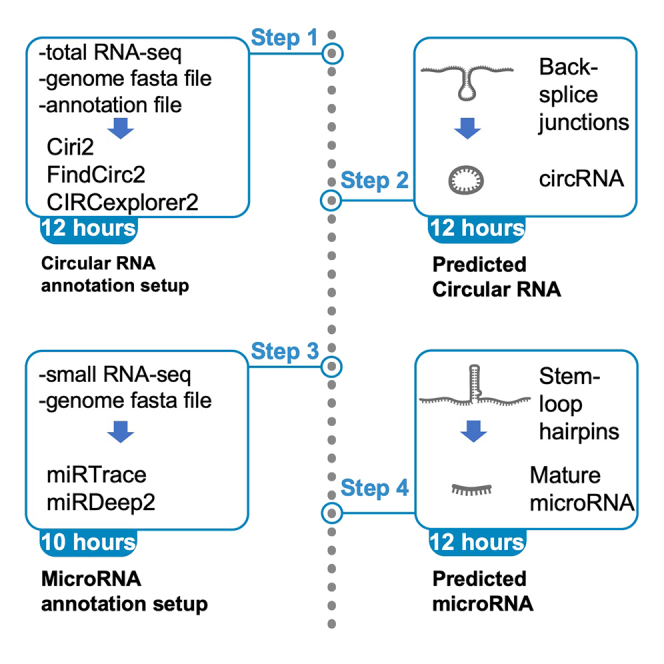

## Before you begin

Non-coding RNAs are implicated in various biological processes including tissue regeneration,^2^ physiological pathways^3^ and disease.^4^ Accurate annotation of non-coding RNAs is therefore a critical initial step in revealing those with functional roles. A plethora of tools exist to annotate and predict non-coding RNAs such as circular RNA (circRNA) and microRNA (miRNA). These tools are either based on organisms with smaller genome size or utilize databases based on genomes of ∼4 Gb or smaller. Herein, I describe a computational framework to predict and annotate non-coding RNAs utilizing the massive 20.3 Gb genome of the Iberian ribbed newt, *Pleurodeles waltl.*^1^ Newts and lungfish typically possess expanded genomes, predominately made-up of repeat elements.^1^^,^^5^ Interspersed embedding of repeat elements can mimic circRNAs that are defined as covalently closed structures lacking a 5′ cap and a 3′ poly(A) tail.^6^ Algorithms to predict miRNAs are particularly prone to false-positives due to mature miRNAs spanning only ∼22 nt in length.^7^ For example, the repeated DNA segments in a large genome can by chance contain regions resembling that of microRNA.

Here, I establish a stepwise framework to annotate circRNAs and miRNAs, utilizing the publicly available newt genome as well as total RNA- and small RNA-sequencing datasets. This analysis builds upon widely cited algorithms allowing for many researchers to easily follow and highlights key modifications required to accommodate large genome. I cover the entire process from acquisition of publicly available data and installation of relevant software to interpretation of the predictions. This protocol can be applied to other species that possess similar annotation challenges.

***Note:*** I have used an Ubuntu operating system with AMD CPU and recommend minimum hardware requirements of 5-TB Hard Drive and at least 128-GB RAM. Alternatively, high-performance computing servers should be considered.

### Software and datasets

1. Download and install Conda <https://anaconda.org/anaconda/conda>, Python <https://www.python.org/downloads/>, R <https://cran.r-project.org/>, and Perl <https://www.perl.org/get.html> as per the software guidelines based on the user’s computer operating system. This protocol was run on a Linux operating system.
2. Download the required dataset from Brown et al. and Elewa et al., see key resources table (KRT).^1^^,^^5^ SRA explorer <https://sra-explorer.info> uses accession numbers of publicly available datasets and provides multiple options for downloading.
3. Download and install relevant software for annotating circRNAs or miRNAs as listed in the key resources table.

**CRITICAL:** Pay attention to the listed versions of the software in the protocol. As not all versions are compatible or lack 64-bit support that is essential for working with large genomes. To note: # denotes a non-executable comment.

## Key resources table

**Table:** 

REAGENT or RESOURCE	SOURCE	IDENTIFIER
**Deposited data**		

PacBio HiFi	Brown et al.^1^	SRA: SRR22542941
*Pleurodeles waltl* genome assemblies	Brown et al.^1^	GenBank: GCA_026652325.1, GCA_031143425.1, GCA_031142525.1
*Pleurodeles waltl* genome assemblies and annotations	Brown et al.^1^	https://doi.org/10.17617/3.90C1ND
*Pleurodeles waltl* transcriptomes	Elewa et al.^5^	BioProject: PRJNA353981, SRA: SRR6001098-SRR6001140

**Software and algorithms**		

bwa-mem v.0.7.17	Li - 1	https://github.com/lh3/bwa
minimap2 v.2.24	Li - 2	https://github.com/lh3/minimap2
agat v.1.0.0	Dainat et al.^8^	https://github.com/NBISweden/AGAT
CIRCexplorer2	Zhang et al.^9^	https://github.com/YangLab/CIRCexplorer2
Find_Circ2	Memczak et al.^10^	https://github.com/rajewsky-lab/find_circ2
CIRI2_v.2.0.6	Gao et al.^11^	https://github.com/bioinfo-biols/CIRI-cookbook/blob/master/source/CIRI2.md
circRNAprofiler v.1.14.0	Aufiero et al.^12^	https://github.com/Aufiero/circRNAprofiler
BEDtools	Quinlan et al.^13^	https://github.com/arq5x/bedtools2
miRDeep2 v.0.1.3	Friedländer et al.^14^	https://github.com/rajewsky-lab/mirdeep2
miRTrace v.1.0.1	Kang et al.^15^	https://github.com/friedlanderlab/mirtrace
bowtie v.1.3.1	Langmead et al.^16^	https://bowtie-bio.sourceforge.net/index.shtml
MAFFT v.7	Katoh et al.^17^	https://www.ebi.ac.uk/jdispatcher/msa/mafft?stype=automatic
GtftoRefflat v.0.1	BIOPET tool suite	https://github.com/biopet/gtftorefflat

**Other**		

Hardware: AMD Ryzen Threadripper PRO 5975WX 32-cores, 256-GB RAM, and Ubuntu v.22.04	N/A	N/A

## Step-by-step method details

### CircRNA annotation using total RNA sequencing datasets

**Timing: 2–3 days**

Here, I describe exact steps to successfully identify and annotate circRNAs.

***Note:*** This protocol creates genome index files and then maps total RNA-seq datasets to the index. Subsequently, it predicts circRNAs using three different published algorithms, performing further downstream analysis on these merged predictions. Based on the reported increased precision of circRNA predictions when combining results from multiple programs, these different algorithms that have been widely-implemented across various species were chosen.^18^ To apply this protocol to other organisms, we require the below listed files at the very least.

 A reference genome fasta file.

Here, I have used the genome that was the output of Pacific Biosciences (PacBio) HiFi sequencing technology as its the highly contiguous genome version for *P. waltl.*^1^ However, a reference genome fasta file outputted from a different sequencing technology such as Nanopore technologies is also compatible.

 Total RNA-seq datasets.

**Figure 1:**
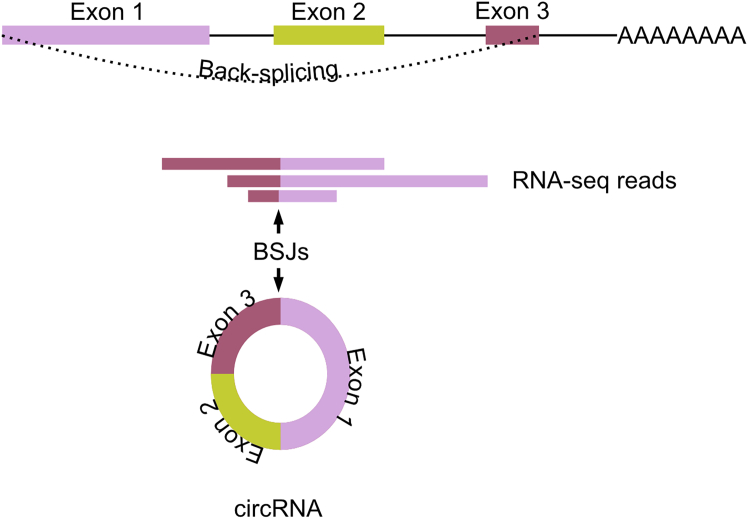
Prediction of circRNAs from total RNA-seq data Detection and quantification of circRNAs in total RNA-seq data is based on reads which overlap non-linear, back-spliced junctions that are formed during circularization of mRNAs. BSJs, back-spliced junctions.

Important to note the type of RNA-seq datasets used to predict the circRNAs. Total RNA-seq datasets employed here are good starting point (see limitations). Total RNA-seq datasets include reads spanning back-splice junctions which are crucial part of the circRNA structure (Figure 1). The circRNA prediction tools utilizes the unique back-splice junction reads to accurately discover circRNAs.

 Annotation file.

A sorted annotation file is required. Sorting of the annotation file could be performed using various tools, such as <agat>. Various circRNA prediction software that are used here, rely on different formats of the annotation files. I have highlighted how to convert the annotation file to the required format at the appropriate steps.

1. Build index of the newt genome <aPleWal1.pri.20220803.fasta>.
  a. Make a working directory with all the required files
    $ mkdir circRNA
    $ cd circRNA
    # <pwd> can be used to identify the current working directory.
    $ mv aPleWal1.pri.20220803.fasta circRNA
    # <mv> moves the required files to the working directory. <cp> can be used to copy instead of move. It is good practice to host all the required files and run the analysis from the working directory.
  b. Install bwa (see key resources table).
  c. Build index (due to large genome size, 128-GB or higher RAM usage is required.)
    # Note that for larger genomes, chromosomes may need to be split.
    $ bwa index -a bwtsw aPleWal1.pri.20220803.fasta
  d. Map the total RNA-seq datasets to the reference genome.
    ***Note:*** Consider performing quality checks on the raw RNA-seq sequencing datasets prior to the analysis. If quality checks indicate there is a large proportion of low quality reads, low quality reads may need to be filtered out. Commands such as <fastqc> (https://www.bioinformatics.babraham.ac.uk/projects/fastqc/) and <multiqc> could be employed. The datasets used here contain relatively high read counts and have undergone prior quality checks.^1^^,^^5^
    i. To run multiple datasets, use bash script as below:
      # Optional, cutadapt or similar tool can be used for adaptor trimming, however, is not essential.
      $ nano circRNA_newt.sh
      # This command opens a bash script where the below code should be then written inside.
      #!/bin/bash
      # Define the reference genome file
      reference="/aPleWal1.pri.20220803.fasta"
      # Total RNA-seq data
      input_files=(
       "SRR6001138_RNA-seq_of_adult_P._waltl_forelimb_stump_0dpa_A"
       "SRR6001133_RNA-seq_of_adult_P._waltl_forelimb_stump_0dpa_B"
       "SRR6001135_RNA-seq_of_adult_P._waltl_regenerating_fore_limb_3dpa_A"
       "SRR6001140_RNA-seq_of_adult_P._waltl_regenerating_forelimb_3dpa_C"
       "SRR6001114_RNA-seq_of_adult_P._waltl_regenerating_forelimb_7dpa_B"
       "SRR6001134_RNA-seq_of_adult_P._waltl_regenerating_forelimb_7dpa_A")
      # Loop through the input files and run bwa mem
      # bwa mem parameters:
      # -T minimum alignment score (default 30), A more permissive setting of 19 is used to capture circRNAs spanning back-spliced junctions that may be underrepresented in total RNA libraries (see limitations).
      # -t is the number of threads utilized. Here its 40 to run the large datasets faster. Should be adjusted based on the available CPUs.
      for file in "${input_files[@]}"; do
       ./bwa/bwa mem -T 19 -t 40 "$reference" "${file}_1.fastq.gz" "${file}_2.fastq.gz" > "${file}.sam"
      done
      # In Linux system, use Ctrl+X, Y and Enter (in this order) to save and close the bash script.
      $ chmod +x circRNA_newt.sh
      $ ./circRNA_newt.sh
2. Use ∗.sam files generated above as input for all three circRNA algorithms.
3. Download the annotation file <aPleWal.anno.v2.20220926.gff3> (see key resources table).

***Note:*** Annotation file can be in numerous formats such as GFF3, GTF, REFFLAT or BED. These files need to be in the format that is compatible with the software being used. Also, the file needs to be sorted. AGAT (see key resources table) could be utilized to perform both, format conversion and annotation file sorting.

# Prediction using Ciri2

$ nano ciri2.sh

#!/bin/bash

# Use AGAT (see key resources table) to sort. Alternatively, IGV sort tools could be used for sorting.

$ agat_convert_sp_gxf2gxf.pl -g aPleWal.anno.v2.20220926.gff3 -o aPleWal.anno.v2.20220926.sorted.gff3

# Use AGAT to convert the annotation file to GTF

$ agat_convert_sp_gff2gtf.pl --gff aPleWal.anno.v2.20220926.sorted.gff3 -o aPleWal.anno.v2.20220926.sorted.gtf

reference="/aPleWal1.pri.20220803.fasta"

annotation="/aPleWal.anno.v2.20220926.sorted.gtf"

# Create an array of your input files

input_files=(

 "SRR6001138_RNA-seq_of_adult_P._waltl_forelimb_stump_0dpa_A"

 "SRR6001133_RNA-seq_of_adult_P._waltl_forelimb_stump_0dpa_B"

 "SRR6001135_RNA-seq_of_adult_P._waltl_regenerating_fore_limb_3dpa_A"

 "SRR6001140_RNA-seq_of_adult_P._waltl_regenerating_forelimb_3dpa_C"

 "SRR6001114_RNA-seq_of_adult_P._waltl_regenerating_forelimb_7dpa_B"

 "SRR6001134_RNA-seq_of_adult_P._waltl_regenerating_forelimb_7dpa_A")

# Loop through the input files and run CIRI2

for file in "${input_files[@]}"; do

 perl CIRI2.pl -T 40 -A "$annotation" -I ".sam${file}" -O "${file}_CIRI.txt" -F "$reference" -G newt_circ2.log

done

$ chmod +x ciri2.sh

$ ./ciri2.sh

# Prediction using CircXplorer2

# First create a virtual environment to run the analysis, using conda

$ conda create -n circenv

# After creating the environment, activate it for the environment to take effect

$ conda activate circenv

# Parse the generated ∗.sam files from Step above, same strategy of running a loop like above can be applied here too.

$ CIRCexplorer2 parse -t BWA /∗.sam > CIRCexplorer2_parse.log

# CIRCexplorer2 uses ∗.refflat annotation format. Use GtftoRefflat to convert to the appropriate format as shown below.

$ java -jar GtftoRefflat-assembly-0.1.jar \

-g /aPleWal.anno.v2.20220926.sorted.gtf \

-r /aPleWal.anno.v2.20220926.sorted.refflat

# Input files as above

$ for file in "${input_files[@]}"; do

  CIRCexplorer2 annotate -r /aPleWal.anno.v2.20220926.sorted.refflat \

  -g /aPleWal1.pri.20220803.fasta \

  -b "/${file}.bed" \

  -o "/${file}_Xplore.txt" > "/${file}_annotate.log"

done

$ conda deactivate circenv

# Prediction using FindCirc2 which uses Pythonv2.7, use below strategy for the rest of the input files.

$ mkdir heart

$ cd heart

$ conda create -n heart python=2.7

$ conda activate heart

$ ./find_circ.py --genome /aPleWal1.pri.20220803.fasta \

--name heart \

/SRR6001112_heart.sam

$ conda deactivate heart

# Once the analysis is complete then deactivate the conda environment as above.

4. Use R package, circRNAprofiler to import the predicted circRNAs from all three circRNA algorithms.

***Note:*** A text output file of the combined and filtered circRNAs is generated and can be saved as a text or a csv file.

5. Follow circRNAprofiler guidelines to merge and perform subsequent statistics <https://github.com/Aufiero/circRNAprofiler>.

***Note:*** There are programs available that can predict *de novo* circRNAs based on reads that appear to map to novel, unannotated splice sites. However, it is recommended to avoid these predictions, particularly in large, repeat element-rich genomes. Predictions that are based on annotation, as described in this protocol, are much more robust.

### miRNA annotation using small RNA sequencing datasets

**Timing:** 2–3 days

Here, I describe exact steps to successfully annotate miRNAs and perform quality-checks.

***Note:*** This protocol generates genome index files and then maps small RNA-seq datasets to that index. Then, it predicts miRNAs and performs further quality checks and downstream post-hoc analysis on the annotated miRNAs. To apply miRNA prediction protocol to other organisms, the below listed files are required at the very least.

 A reference genome fasta file.

As mentioned earlier, the reference genome fasta file is compatible regardless of the technology used to generate the genome fasta file.

 Small RNA-seq datasets.

Unlike, total RNA-seq used for circRNA prediction, the small RNA-seq datasets are suitable for miRNA predictions. They contain reads corresponding to the length of mature miRNAs but may also contain various other small RNAs such as siRNA, piwiRNA, tRNA fragments. Therefore, quality checks and filtering steps are highlighted in the protocol which optimize these datasets for miRNA discovery.

6. Build index of the newt genome <aPleWal1.pri.20220803.fasta>.
  a. Install Bowtie v.1.3.1 that supports 64-bit (see key resources table).
  b. Build index (due to large genome size, 128-GB or higher RAM usage is required.).

$ bowtie-build --large-index aPleWal1.pri.20220803.fasta Unsplit_newt

# Un-split genome is preferrable. Creating numerous small computational fragments of chromosomes could cause issue at later stages (see problem 1). Ideal to use options such as –large-index to accommodate large genomes.

7. Run quality checks on small RNA-seq datasets using miRTrace.

***Note:*** MiRTrace performs quality checks, filters and reports on read length distribution, RNA type, contamination, etc., in addition to the adapter removal.

***Optional:*** The miRTrace outputs a html report summarizing all the quality checks it performed. This html report is interactive and contains information regarding what classifies as good or bad metric. The html report for this protocol can be downloaded from Brown et al.^1^ (Data S6).

8. Map the output from miRTrace to the genome.

# Create Config_fa.txt as per the command below and use Config_fa.txt to list multiple data files used.

# miRBase database lacks support for salamanders, as a next best choice, use xenopus instead.

$ mirtrace qc --species xtr --adapter TGGAATTCTCGGGTGCCAAGG \

SRR6001100_small_RNA-seq_of_P._waltl_late_embryo.fastq.gz \

SRR6001101_small_RNA-seq_of_P._waltl_larvae.fastq.gz \

SRR6001102_small_RNA-seq_of_P._waltl_oocytes.fastq.gz \

SRR6001104_small_RNA-seq_of_adult_P._waltl_heart.fastq.gz \

SRR6001105_small_RNA-seq_of_adult_P._waltl_lung.fastq.gz \

SRR6001107_small_RNA-seq_of_adult_P._waltl_eyes.fastq.gz \

SRR6001127_small_RNA-seq_of_adult_P._waltl_brain.fastq.gz \

SRR6001128_small_RNA-seq_of_adult_P._waltl_regenerating_forelimb_7dpa.fastq.gz \

SRR6001130_small_RNA-seq_of_adult_P._waltl_liver.fastq.gz \

SRR6001103_small_RNA-seq_of_adult_P._waltl_regenerating_forelimb_3dpa.fastq.gz --write-fasta

# Above command outputs a folder:qc_passed_reads.all.collapsed containing the quality checked fasta files that is used for downstream analysis.

# Make a Config_fa.txt file to run numerous collapsed fasta datasets together.

$ echo "qc_passed_reads.all.collapsed/SRR6001100_small_RNA-seq_of_P._waltl_late_embryo.fasta S00

qc_passed_reads.all.collapsed/SRR6001101_small_RNA-seq_of_P._waltl_larvae.fasta S01

qc_passed_reads.all.collapsed/SRR6001102_small_RNA-seq_of_P._waltl_oocytes.fasta S02

qc_passed_reads.all.collapsed/SRR6001103_small_RNA-seq_of_adult_P._waltl_regenerating_forelimb_3dpa.fasta S03

qc_passed_reads.all.collapsed/SRR6001104_small_RNA-seq_of_adult_P._waltl_heart.fasta S04

qc_passed_reads.all.collapsed/SRR6001105_small_RNA-seq_of_adult_P._waltl_lung.fasta S05

qc_passed_reads.all.collapsed/SRR6001107_small_RNA-seq_of_adult_P._waltl_eyes.fasta S07

qc_passed_reads.all.collapsed/SRR6001127_small_RNA-seq_of_adult_P._waltl_brain.fasta S27

qc_passed_reads.all.collapsed/SRR6001128_small_RNA-seq_of_adult_P._waltl_regenerating_forelimb_7dpa.fasta S28

qc_passed_reads.all.collapsed/SRR6001130_small_RNA-seq_of_adult_P._waltl_liver.fasta S30" > Config_fa.txt

***Note:*** The miRTrace output contains filtered small RNA-seq reads. To get more info regarding each parameter used in mapper.pl please refer to miRDeep2 manual (see key resources table). The command below takes in the filtered small RNA-seq fasta files as listed in the “Config_fa.txt” and maps to the indexed genome “Unsplit_newt”. Resulting in “reads_vs_genome.arf” containing the mapped reads. This output file is in .arf format and is used in the downstream miRDeep2 analysis.

$ mapper.pl Config_fa.txt -d -c -i -j -l 18 -m -o 18 -p Unsplit_newt -t reads_vs_genome.arf -v -n

9. Run miRDeep2 to predict miRNAs from the above outputs.
  a. Extract miRTrace output “/all_like.fasta” containing all the filtered collapsed reads from the small RNA-seq used available as fasta format.
  b. Download both, precursor and mature miRNA sequences from miRBase or similar database. Use closely related species when species of interest is missing.

$miRDeep2.pl all_like.fasta aPleWal1.pri.20220803.fasta reads_vs_genome.arf newt_MAT3.fasta output_xenfasta newt_PRE3.fasta 2>mirdeep.log

***Note:*** Above is the final command that predicts miRNAs. Refer to miRDeep2 manual (see key resources table) for in-depth information on the parameters used and the numerous output files that miRDeep2 generates. In brief, this command utilizes the quality checked miRTrace output (all_like.fasta), the genome (aPleWal1.pri.20220803.fasta), the mapped reads of the combined small RNA-seq datasets (reads_vs_genome.arf), any known mature miRNA sequences (newt_MAT3.fasta) and precursor miRNA sequences (newt_PRE3.fasta) of the species (*P. waltl* in this case) and mature miRNA sequences from the related species (output_xenfasta, *X. tropicalis* in this case).

***Optional:*** The miRDeep2 also outputs a html report among various other in-depth reports. For more info on the detailed outputs, please refer to miRDeep2 manual (see key resources table). This html summarizes the miRNA prediction and explains the parameters. The html report for this protocol is available in Brown et al.^1^ (Data S7).

10. Save data generated above in a new folder to compare with a re-run of miRDeep2 as listed below.

**CRITICAL:** Find prepare_signature.pl in miRDeep2 and change from

system ("bowtie -f -v $read_align_edit_distance -a --best --strata --norc $dir/precursors.ebwt $file_reads $dir/reads_vs_precursors.bwt 2> /dev/null");

**Figure 2:**
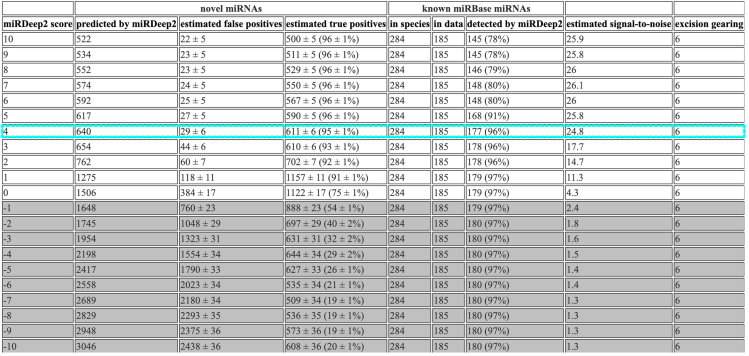
Predicted miRNAs report output from miRDeep2 Row highlighted in blue indicates a miRDeep2 score cut-off that represents a strong signal-to-noise ratio.

**Figure 3:**
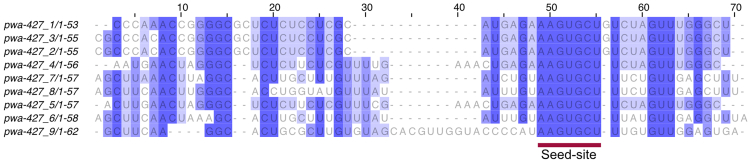
Multiple alignment analyses of duplicated miRNA precursor sequences This example MAFFT alignment of 9 duplicated miR427/430/302 precursor sequence shows a strong conservation at the seed-site, AAGUGCU. Dark purple regions indicate conservation of bases. This conservation pattern suggests genuine duplication of miRNAs where the seed-site is preserved. The seed-site is the most important sequence of the miRNA that aids in binding to its target. MAFFT, multiple alignment using fast Fourier transform.

**Figure 4:**
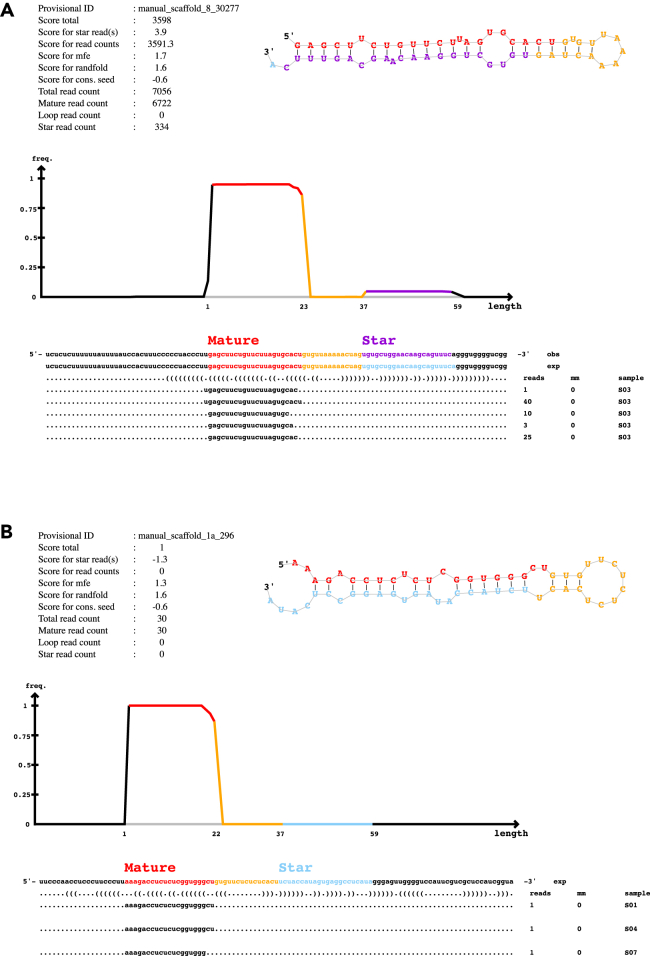
miRNAs detected by miRDeep2 PDFs output from miRDeep2 provide detailed information on individual miRNAs detected that can be used to manually check miRNA prediction quality. (A) Example of a high-quality miRNA prediction which has read coverage of both the mature and star sequences, with high total read counts originating from multiple samples. (B) Example of a low-quality miRNA prediction with low total reads from few samples and no coverage of the miRNA star sequence. Score details and read counts are provided in the top left table, the predicted hairpin structure is shown in the top right. The middle graph represents read distribution in the miRNA precursor and each unique read and the sample it originated from is listed below.

to.

system ("bowtie -f -v 0 -a --best --strata --norc $dir/precursors.ebwt $file_reads $dir/reads_vs_precursors.bwt 2> /dev/null");

**CRITICAL:** The above change affects the bowtie alignment steps that ensures that there’s no mismatch when aligning the sequences. This is done by setting <-v 0>. This step ensures that only perfectly aligned matches are carried over to the miRDeep2.pl which is the next and the final step of miRNA prediction.

11. Re-run miRDeep2 with more stringent settings to counter false-positive due to numerous repeat regions in the newt genome.

$ miRDeep2.pl all_like.fasta aPleWal1.pri.20220803.fasta reads_vs_genome.arf newt_MAT3.fasta output_xenfasta newt_PRE3.fasta 2>mirdeep_Strict.log

***Note:*** Above is the same command as Step 9b. However, in this run one expects to see a reduction in the overall number of miRNAs compared to earlier. This reduction is due to some of the miRNA sequences being discarded because of the prior alteration of prepare_signature.pl.

***Optional:*** In large genomes with repeated regions, there’s a higher chance of multimapping of the predicted miRNAs. This inflates the total number of predicted miRNAs. MiRDeep2 prediction automatically discards any miRNA that has more than five alignments. To be stringent, the below steps can be employed to further reveal miRNAs with various degree of unique reads.

12. From miRDeep2 output, choose the miRNAs with a higher signal to noise ratio (Figure 2).

***Note:*** Signal-to-noise ratio is calculated by taking total predicted miRNA hairpins over estimated false positive miRNA hairpins. A good miRDeep2 run would reflect higher signal (true predicted miRNA candidates) over the noise (false positive miRNAs), usually 10:1 is considered very good.^14^ For the newt data, the miRDeep2 score cut-off is 4 (Figure 2).

13. Build a bowtie index out of the selected miRNAs.

$ bowtie-build miRs_855.fasta miRs_855_index

14. Align the indexed generated above to the miRTrace output used above.

$ bowtie --best --strata -v 0 -m 1 --al output -p 10 -f miRs_855_index all_like.fasta > Aligned_855.txt

***Note:*** The values for -v and -m used above are not required to run the code. Yet, the inclusion of the parameters in the above code is to imply that these are adjustable based on stringency of the alignment required. For more info on bowtie see key resources table.

15. Use miRs with the repeated precursor sequences to run multiple alignment using fast Fourier transform (MAFFT) tool <https://www.ebi.ac.uk/jdispatcher/msa/mafft?stype=automatic> to align similar miRNA precursors.
16. Use online MAFFT to visualize the alignment, percentage identity or phylogram.

***Note:*** Can also download the output in FASTA format for further visualization of the repeated precursor sequences. A good alignment of a genuine duplicated miRNA should preserve the seed-site sequence (Figure 3).

***Optional:*** There could be various precursor sequences that uniquely mapped to the genome but still resulted in identical mature sequences. Above steps are for further alignment and visualization of such identical mature sequences. In Figure 3, repeated miR-427/430/302 precursor sequences are aligned which reveals a preservation of the seed-site. Seed sequence of the miRNA is the most important aspect of miRNA:mRNA target binding.

17. Use secondary structures, mature and star read counts along with miRDeep2 scores to investigate miRNA of interest (Figure 4). The PDF files of each miRNA candidate is available in the miRDeep2 output folder labeled as “PDFs”.

***Note:*** The timing listed for both circRNA and miRNA annotation protocols are based on the newt genome used. This will be significantly longer or shorter dependent on size of the genome and transcriptomic data used.

## Expected outcomes

The circRNA protocol utilizes three prediction algorithms which outputs either a text file or a BED format file. These files are then loaded into circRNAprofiler (R package, see key resources table). The tutorial <https://bioconductor.org/packages/devel/bioc/vignettes/circRNAprofiler/inst/doc/circRNAprofiler.html> contains in-depth quality checks and guides to visualize the predicted circRNAs. Researchers can further investigate the involvement of repeat elements in the biogenesis of circRNA. This can be done by intersecting the final output of the circRNAprofiler text file, which contains all the merged circRNAs, with the repeat element annotation GTF file, using BEDtools. The intersected result will reveal any pattern in relation to repeat elements and circRNAs. Subsequent statistical analysis of this pattern will highlight the degree of repeat element association with the circRNAs in a given species.

The miRNA annotation protocol results in miRDeep2 outputs (see key resources table). The result.html file shows the metrics of the miRNA run and hovering on the title reveals further information. Other outputs such as result.bed and result.csv can further be utilized to download the precursor, mature or star sequences. The precursor sequences of the miRNAs of interest can then be used for further MAFFT alignment (Figure 3) and drawing phylograms. In addition, the PDFs folder contains secondary structures and read count summary of each miRNA candidate that can be further investigated (Figure 4). Researchers can further intersect the result.bed with repeat element annotation file to reveal the numbers of miRNAs embedded in repeat elements in the given species. The result.bed file can also be utilized to compare miRNA predictions from other tools.

Both circRNA and miRNA location from the final output can be utilized to visualize distribution across the genome using relevant tools as shown in Brown et al.^1^

## Limitations

The accuracy of the circRNA annotation protocol is dependent on the read depth and quality of transcriptomic and genomic data used. A read depth of over ∼100 million is generally considered good. As the splice-sites serve an important role in the biogenesis of circRNAs, the quality of the species’ annotation further aids in the accuracy of the prediction. Total RNA-seq datasets have widely been used to predict circRNAs and is an ideal initial step when this data is already available, however there is potential that some reads mapping to back-spliced junctions may not originate from circRNAs but from other events such as mRNA trans-splicing or template-switching during PCR amplification of sequencing libraries. This could be mitigated by targeted circRNA sequencing approaches where RNA is first treated with RNase R, depleting linear mRNA and highly enriching for circRNA, minimizing the detection of false positives in sequencing data.

The miRNA annotation protocol benefits from a highly contiguous genome and multiple small RNA-seq datasets. This is reflective in the signal-to-noise ratio (above 10:1 is considered good) and corresponding miRDeep2 scores. The identification of potential novel miRNA candidates is further highlighted in the protocol. However, caution should be applied when investigating these novel miRNA candidates. In large genomes filled with repeat elements such as of salamanders and lungfish, repetitive sequences can be misannotated as potential miRNAs. Given the strong conservation of miRNAs across species, a feasible post-hoc test of checking homology with existing miRNAs is highly recommended. For the novel miRNA candidates, higher stringency such as checking for multimapping or detection via stem-loop primers in a PCR reaction should be considered. Alternatively, complementing the miRDeep2 miRNA predictions with other tools can also be applied as performed in Brown et al.^1^

## Troubleshooting

### Problem 1

Issues relating to software installation (related to Step 1).

### Potential solution

Most likely error arises due to either missing dependency or a mismatch in the versions. Create an environment using Conda <https://docs.conda.io/projects/conda/en/latest/index.html> or Mamba <https://mamba.readthedocs.io/en/latest/index.html>. In the newly created environment, install the required dependencies and the appropriate versions that are required. As shown in Step 2.

### Problem 2

Issues relating to building index or working with large genomes (related to Steps 1b, 5b and 8).

### Potential solution

Computational tools are usually not designed to accommodate giant genomes such as newt with its 20.3 Gb genome. Due to this large size, a lot of algorithms, especially the ones lacking 64-bit support crashes constantly. Please pay attention to the versions mentioned here as they have been tested to work with a large genome. For step 8, miRDeep2 has a built-in bowtie but is an older version that lacks support for large genomes. A straightforward approach to rectify this is to delete the bowtie version that is installed with miRDeep2. Set the bowtie v1.3.1 as the default version on your system. This enables the correct version of bowtie to be used for the entire miRNA prediction run. Another option is to computationally divide the chromosomes based on size thresholds set by these algorithms. Please note that the first four chromosomes of the newt genome are already split, and additional chromosomes may require splitting. Caution should be applied when splitting the genome as multiple splits of the genome can lead to further downstream issues. One example from various computationally created chromosomes is that prediction of potential miRNAs close to split sites could be disrupted.

### Problem 3

Failure to merge any or limited circRNA predictions in circRNAprofiler (related to Step 3).

### Potential solution

It is unlikely that there’s no overlap in the circRNA prediction algorithms. Predictions from one or more tools failing to overlap could be due to how these individual tools annotate and report ‘Start’ location of the circRNA. Please take note of the ‘Start’ or ‘End’ location of the circRNAs, if there’s a difference of one or two base pairs then this may lead to that circRNA from being excluded despite being the same circRNA. Please subtract or add, depending on the pattern, one base pair to all circRNAs therefore allowing them to have same Start sites. This particular issue is resolved in the current version of circRNAprofiler, for more info consult the relevant GitHub (see key resources table).

### Problem 4

Output of miRTrace causes error when running mapper.pl or any downstream analyses (related to Step 7).

### Potential solution

The output of miRTrace contains quality checks of the small RNA-seq datasets and a FASTA format file of all collapsed reads. This step also clips adaptors of the small RNA-seq datasets and prior adaptor trimming is not required. The collapsed reads may contain whitespaces or unwanted terms in the header, such as “rnatype:mirna” and this is the most likely reason for mapper.pl to fail. This issue could also be encountered elsewhere so please pay attention to unwanted characters or whitespaces. This could be fixed using a sed command/s for example.

$ sed ‘s/ .∗//’ # delete any terms after a whitespace.

$ sed ‘s/ /_/g’ # replace it with underscore instead of deletion.

### Problem 5

Unable to implement known mature or precursor miRNAs for a given species in the miRDeep2 run (related to Step 8).

### Potential solution

The accuracy of miRDeep2 miRNA prediction is improved when provided with known mature and precursor miRNA sequences of the species of interest. If one is working with species with no prior knowledge, then please provide the sequences from the closest related species. Other errors such as the format of mature and precursor sequences could lead to failure in recognition by miRDeep2. Both, mature and precursor sequences used in this protocol were capitalized and in DNA format.

## Resource availability

### Lead contact

Further information and requests for resources and reagents should be directed to and will be fulfilled by the lead contact, Ketan Mishra (ketan.mishra@ki.se).

### Technical contact

Technical questions on executing this protocol should be directed and will be answered by the technical contact, Ketan Mishra (ketan.mishra@ki.se).

### Materials availability

This study did not generate new unique reagents.

### Data and code availability

Genome and annotation files are available through the Max Planck Digital Library <https://doi.org/10.17617/3.90C1ND> and through NCBI under BioProject PRJNA847026. Transcriptomic datasets are deposited under BioProject PRJNA353981 and BioSample accession SAMN07571895, Runs SRR6001098-SRR6001140.

## Acknowledgments

I thank András Simon for the valuable discussion and funding. Computation and storage resources were provided by the Swedish National Infrastructure for Computing (SNIC) at UPPMAX, funded by the 10.13039/501100001862Swedish Research Council through grant agreement no. 2018-05973. The work was funded by the 10.13039/501100001862Swedish Research Council (2023-02820), 10.13039/501100002794Cancerfonden (22 2424 Pj), the 10.13039/100010663European Research Council (951477), the 10.13039/501100004063Knut and Alice Wallenberg Foundation (project grant 2018-2022), and the 10.13039/501100004047Karolinska Institute to András Simon.

## Author contributions

K.M., conceptualization, methodology, software, investigation, formal analysis, and writing.

## Declaration of interests

The author declares no competing interests.
